# Enabling Quick Response to Nitrogen Dioxide at Room Temperature and Limit of Detection to Ppb Level by Heavily n-Doped Graphene Hybrid Transistor

**DOI:** 10.3390/molecules28135054

**Published:** 2023-06-28

**Authors:** Si-Wei Song, Qian-Min Wang, Miao Yu, Zhi-Yuan Tian, Zhi-Yong Yang

**Affiliations:** School of Chemical Science, University of Chinese Academy of Sciences, 19A Yuquanlu, Beijing 100049, China

**Keywords:** nitrogen dioxide, graphene, sensor, field effect transistor, poly(p-phenylene vinylene)

## Abstract

Sensitive detection of nitrogen dioxide (NO_2_) is of significance in many areas for health and environmental protections. In this work, we developed an efficient NO_2_ sensor that can respond within seconds at room temperature, and the limit of detection (LOD) is as low as 100 ppb. Coating cyano-substituted poly(p-phenylene vinylene) (CN-PPV) films on graphene (G) layers can dope G sheets effectively to a heavy *n* state. The influences of solution concentrations and annealing temperatures on the *n*-doping effect were investigated in detail. The CN-PPV–G transistors fabricated with the optimized parameters demonstrate active sensing abilities toward NO_2_. The *n*-doping state of CN-PPV–G is reduced dramatically by NO_2_, which is a strong *p*-doping compound. Upon exposure to 25 ppm of NO_2_, our CN-PPV–G sensors react in 10 s, indicating it is almost an immediate response. LOD is determined as low as 100 ppb. The ultrahigh responding speed and low LOD are not affected in dry air. Furthermore, cycling use of our sensors can be realized through simple annealing. The superior features shown by our CN-PPV–G sensors are highly desired in the applications of monitoring the level of NO_2_ in situ and setting immediate alarms. Our results also suggest that transfer curves of transistors can react very promptly to the stimulus of target gas and, thus, are very promising in the development of fast-response sensing devices although the response values may not reach maximum as a tradeoff.

## 1. Introduction

Nitrogen dioxide (NO_2_) is notorious for its severe threats to our health and environment. For instance, our teeth, respiratory and cardiovascular systems could be hurt seriously by low concentration NO_2_, and the acid rain formed by NO_2_ dissolving in water leads to detrimental damages to both the aquatic and terrestrial ecosystems [1]. Therefore, great efforts have been devoted to fabricating efficient sensors that can quickly respond to a low level of NO_2_. Other properties of NO_2_ sensors, such as operation temperature, reliabilities and recovery time, are also very important for realizing successful applications.

Quite a lot of material was used in NO_2_ sensing. Semiconducting metal oxides (MOXs) demonstrate excellent sensitivity in NO_2_ detection [1,2]. However, most of them need work at above room temperature (RT) [3,4]. Studies on graphene (G) materials stimulated the rapid developments of other two-dimensional (2D) materials [5]. Very soon these materials were tested as NO_2_-sensing candidates because of their outstanding electrical properties, very high surface-to-volume ratio, excellent stability and flexibility [3,6,7,8,9,10,11]. Among all of the 2D sheets, preparation of G materials is most convenient due to the varied, well-developed synthesis approaches with low cost. Reduced G oxides (RGOs) are produced mainly in solution, which facilitates their compounding with nano MOXs that are prepared usually in liquid as well. Thus, numerous MOX–RGO hybrids were fabricated with the anticipation of combining the advantages of two kinds of components, including ZnO–RGO, SnO_2_–RGO, Cu_2_O–RGO [12,13,14,15,16]. Most of MOX–RGO films demonstrate improved sensing performances in one or several aspects. Besides inorganic components, organic compound–RGO or GO hybrids, such as dopamine, poly(styrene sulfonic acid) sodium salt and amyloid nanofibril, manifest remarkable NO_2_ sensing capabilities as well [17,18,19]. RGO with other 2D materials, mainly MoS_2_, can also realize quick response or RT operation [20,21]. In comparison with GO or RGO materials, G layers fabricated through chemical vapor deposition or mechanical cleavage from highly oriented pyrolytic graphite have better uniform electronic properties. The sensing capabilities of pristine G layers can be promoted by assistive approaches, for instance, post modification by O_3_ or lithography, or under UV illumination or heating [3,10,22,23,24,25]. Most current NO_2_ sensors rely on the changing of resistance. In contrast to the widely studied resistance devices, NO_2_ sensors based on field effect transistor (FET) have not been paid enough attention although the detection of a single NO_2_ molecule was reported on a G FET [26]. Gate bias (V_g_) in FET is another external option for tuning the sensor activities besides the above mentioned illumination or heating, etc. Poly(p-phenylene vinylene) (PPV) is a kind of conjugated polymer. Its derivatives were fabricated into in kinds of electronic devices, such as FET, solar cells and light-emitting diodes [27,28]. In addition to electronics, they were used in gas detecting areas after forming hybrid films with other materials including porous silicon, carbon nanotubes, ZnO nanorods [27,28].

Here, we report an effective NO_2_ sensor that can respond to 25 ppm of target molecules within 10 s at room temperature, and the limit of detection (LOD) is as low as 100 ppb. Quick response and low LOD are not affected in dry air. Furthermore, cycle use of our sensors can be realized through simple heating treatment. Coating cyano-substituted poly(p-phenylene vinylene) (CN-PPV) on G sheets shifts the minimum point (MP) of FET transfer curves of hybrid films to a very negative V_g_ value, revealing the giant *n*-doping effect of CN-PPV to G (n-doping effect means the Fermi level of G is shifted toward above the G Dirac point). NO_2_ is well known for its strong *p*-doping abilities to G layers (p-doping effect means the Fermi level of G is shifted toward below the G Dirac point). As a consequence, exposing to NO_2_ leads to the dramatic reduction of the *n*-doping state of CN-PPV–G hybrid films. The transfer curves of transistors, usually referring as the variation of the current (I) between drain (d) and source (s) electrode with V_g_, are able to sensitively collect the changing of the G doping state before and after NO_2_ exposure. Thus, relying on the vigorous alterations of the transfer curves of CN-PPV–G transistors, efficient NO_2_ detection with high responding speed is realized. The results in this report demonstrate the promising detection abilities based on the field effect of 2D materials and provide valuable supports for developing new types of high performance gas sensors.

## 2. Results and Discussions

### 2.1. Structural Characterizations of CN-PPV–G Films

**Figure 1:**
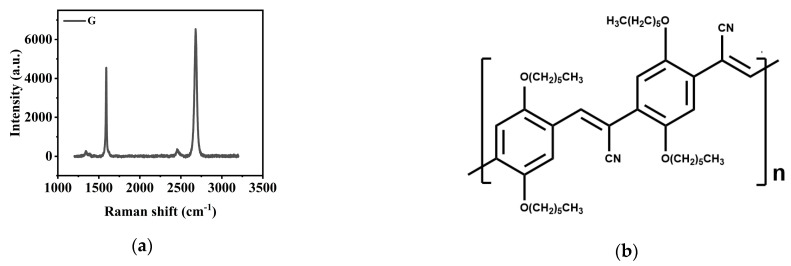
(**a**) Raman spectroscopy of G sheets; (**b**) illustration of chemical structure of CN-PPV.

**Figure 2:**
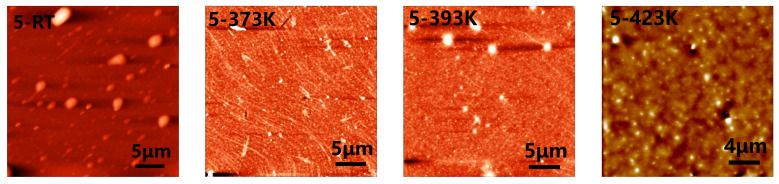
AFM topography images of CN-PPV–G films prepared with the solution of 5 mg mL^−1^ before and after 1 h thermal annealing at 373 K, 393 K and 423 K.

**Figure 3:**
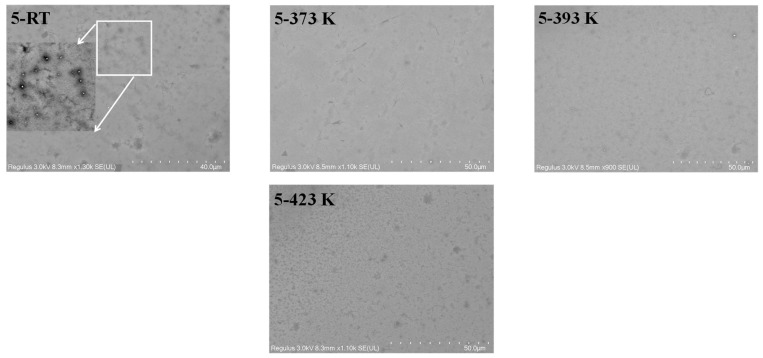
SEM images of CN-PPV–G films prepared with the solution of 5 mg mL^−1^ before and after 1 h thermal annealing at 373 K, 393 K and 423 K. Contrast and brightness of zoom-in images were post adjusted to clearly show the topographical target, which is not easily recognized in the small-size original micrographs.

G sheets after transfer onto SiO_2_/Si substrate were characterized by Raman spectroscopy (Figure 1a). The spectroscopy shows typical G and 2D signals. The D peak at 1350 cm^−1^, indicating defects and fractures of G layers, is rather weak, suggesting the quality of G layers is very high. The average thickness estimated from the G and 2D peaks is around 1–2 layers, which is further confirmed by the following characterizations of atomic force microscopy (AFM, Figure 2 and Figure S1) and scanning electron microscopy (SEM, Figure 3 and Figure S2). In many practical applications of G electronic devices, the rigid single layer of G may not show best performances because its structure is more easily destroyed during the transfer and device fabrication processes and its electronic properties tend to be sensitively affected by the charges trapped at the interface of substrates. Therefore, multilayer G sheets were used. Highly conductive FET devices, showing intrinsic properties of pristine G sheets, were acquired after thermal annealing at 423 K.

The doping effect of CN-PPV (Figure 1b) to G is related to both solution concentration and temperature of thermal annealing. Therefore, the CN-PPV–G films prepared from different concentrations and annealed at varied temperatures were characterized in detail. Surface topography of CN-PPV–G hybrid films was firstly investigated through AFM (Figure 2 and Figure S1). After casting CN-PPV film from low concentration solution (0.01 mg mL^−1^, Figure S1a), G ripples and wrinkles, which appear as thin bright lines, and hexagonal bilayer areas can still be recognized in AFM images, indicating the CN-PPV film is extremely thin and may not cover the whole surface of G. Thermal annealing at 373 K, 393 K and 423 K has no obvious effect on forming continuous film. Increasing the concentration to 1 mg mL^−1^ (Figure S1b), ripples and wrinkles of G sheets are not completely covered yet. When the solution was further promoted to 5 mg mL^−1^ (Figure 2), the information from G sheets had totally disappeared. Meantime, large clusters were observed in the RT samples. Dense, continuous film with much lower surface roughness was formed via thermal treatments. With the largest concentration (20 mg mL^−1^), thick film was formed in the as-casted samples (Figure S1c). The morphology of this sample was smoothed apparently through thermal treatments as well. From the AFM characterizations on the samples prepared from different concentrations and annealed at three temperatures, it can be seen that thermal annealing has obvious effects on improving the qualities of CN-PPV films fabricated from dense solutions (5 mg mL^−1^ and 20 mg mL^−1^) because large clusters were reduced and films were smoothed apparently through annealing.

SEM micrographs collected on the CN-PPV–G films give similar results with AFM data overall. Only very thin CN-PPV film is formed at low concentrations (0.01 mg mL^−1^ and 1 mg mL^−1^) as the multilayer areas (some are pointed out by arrows in Figure S2a,b) are disclosed clearly. For the RT samples cast from dense solutions (5 mg mL^−1^ and 20 mg mL^−1^), large clusters appear (see the inserted zoom-in images), and films are very rough (Figure 3 and Figure S2c). Annealing treatments have a significant effect on improving the qualities of these films as it can be seen that both clusters and surface roughness are apparently reduced.

**Figure 4:**
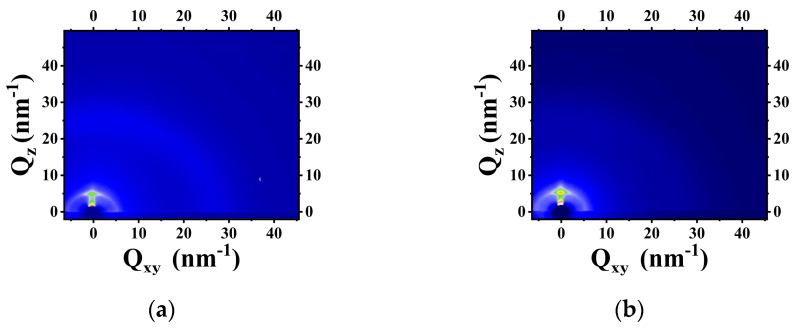
(**a**,**b**) GIWAXS patterns collected on CN-PPV–G films prepared from 5 mg mL^−1^ before and after annealed 1 h at 423 K, respectively. The first dot in the *z* direction of each image is not a signal that can be reasonably generated by our CN-PPV–G films, so we discarded the corresponding peak in the fitting profiles (Figure S4).

After finishing the analysis on the surface topography of CN-PPV–G samples through AFM and SEM imaging, the stacking of CN-PPV was probed further by grazing-incident wide angle X-ray scattering (GIWAXS) technique. GIWAXS images (Figure 4 and Figure S3) have no observable patterns in the *xy* direction, suggesting CN-PPV lacks long-range ordered in-plane arrangement; while in the *z* direction, diffraction signals are rather clear. In the fitting profiles (Figure S4), the peak (*P*_1_) corresponding to the second dot in the *z* direction of GIWAXS patterns suggests an interplanar distance of 1.9 nm, implying part of CN-PPV adopts an edge-on orientation in the out of plane direction. (The first dot in the *z* direction of each pattern is not a signal that can be reasonably generated by our CN-PPV–G films, so we discarded the corresponding peak in the fitting profiles). The GIWAXS characterizations suggest CN-PPV films are quasi amorphous, and post annealing treatments have no observable effect on forming ordered arrangement. The Raman spectroscopy of thin CN-PPV–G films prepared from 0.01 mg mL^−1^ solution detects the signals from both G substrate and CN-PPV component (Figure S5). Multi peaks generated by CN-PPV are rather complicated. Some of them overlap with the peaks of G substrate. When the thickness of CN-PPV film increases with the solution concentration, its strong fluorescence makes Raman analysis unfeasible because the Raman signals are drowned in background completely.

### 2.2. Doping Effect of CN-PPV to G Layers with Different Solution Concentrations and Annealing Temperatures

**Figure 5:**
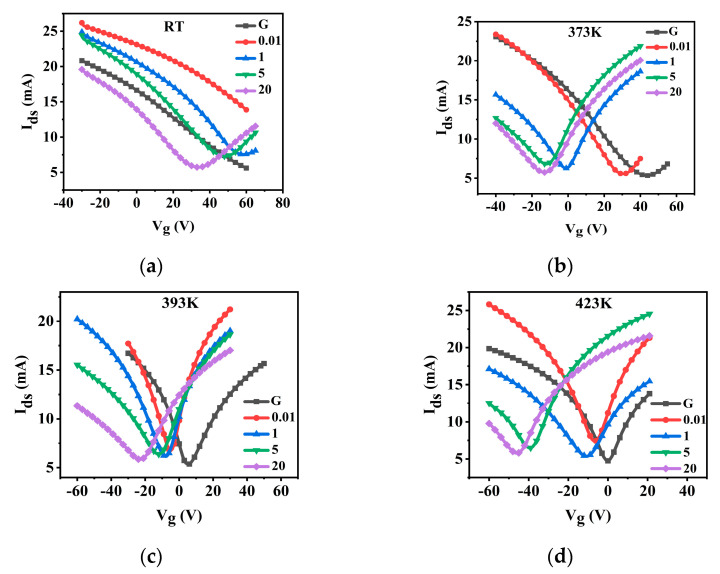
Transfer curves (V_ds_ = 1 V) of pristine G and CN-PPV–G samples casted from different concentrations before (**a**) and after annealing 1 h at 373 K (**b**), 393 K (**c**) and 423 K (**d**).

**Figure 6:**
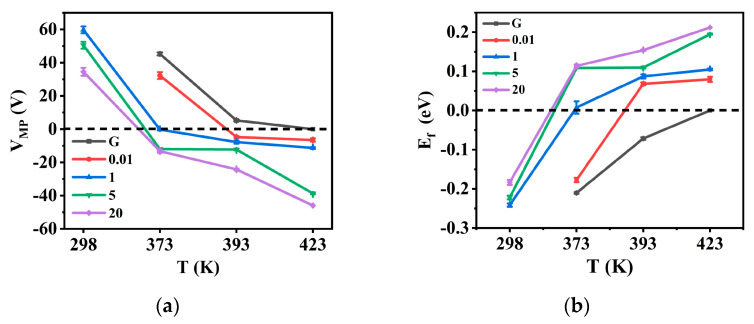
(**a**) Summarized V_g_ of MP on the transfer curves and (**b**) E_f_ (related to the MP position) of pristine G and CN-PPV–G samples casted from different concentrations before and after annealing 1 h at different temperatures. The dashed line in (**a**,**b**) indicates the V_g_ = 0 and energy level of MP (set as reference point of E_f_), respectively. RT is around 298 K.

CN-PPV has a significant *n*-doping effect to G sheets (Figure 5). Before annealing, MP on the transfer curves of pristine G sheets and CN-PPV–G prepared from 0.01 mg mL^−1^ solution had not been detected even at V_g_ = 60 V (Figure 5a), which means the Fermi level (E_f_) of these two samples is lower than 0.252 eV (the E_f_ position is estimated to be at 0.252 eV below MP if V_g_ = 60 V, according to the equation given in the experimental section). When the concentration of solution increases from 1 mg mL^−1^, 5 mg mL^−1^ to 20 mg mL^−1^, MP gradually shifts from around V_g_ = 60 V, V_g_ = 51 V to V_g_ = 35 V, respectively (Figure 5a and Figure 6a), revealing the up-movement of E_f_ (Figure 6b) and *n*-doping tendency of CN-PPV to G sheets. At V_g_ = 0 V, I_ds_ reading from the transfer curves in Figure 5a decreases as I_ds_ (0.01 mg mL^−1^) > I_ds_ (1 mg mL^−1^) > I_ds_ (5 mg mL^−1^) > I_ds_ (G) > I_ds_ (20 mg mL^−1^). The I_ds_ values in the output data obtained at V_g_ = 0 V (Figure S6a) confirm this tendency perfectly. Before annealing, all the samples are in *p*-doing state.

After annealing at 373 K, MP of all samples was displaced to a left position in comparison with the corresponding one in the as-casted samples because of the desorption of *p*-doping substances such as O_2_ and H_2_O in the films (Figure 5b and Figure 6a). MP of G and CN-PPV–G casted from 0.01 mg mL^−1^ solution is at V_g_ = 45 V and V_g_ = 32 V, respectively, implying E_f_ of these two samples is still much lower than the MP position (Figure 6b). E_f_ of the CN-PPV–G deposited from 1 mg mL^−1^ solution is roughly at MP position. For the samples from denser solutions, their MPs are located at negative V_g_ range (Figure 5b and Figure 6a), revealing their E_f_ locales above MP (Figure 6b). Thus, their *n*-doping state is disclosed clearly. I_ds_ values given by the transfer (Figure 5b) and output curves (Figure S6b) demonstrate the same sequence: I_ds_ (G) > I_ds_ (0.01 mg mL^−1^) > I_ds_ (5 mg mL^−1^) > I_ds_ (20 mg mL^−1^) > I_ds_ (1 mg mL^−1^). After annealing at 373 K, CN-PPV–G (1 mg mL^−1^) is a critical point (E_f_ roughly overlapping with MP), the samples prepared from denser solution are in a *n*-doping state whereas pristine G and CN-PPV–G from lower concentration (0.01 mg mL^−1^) are still in a *p*-doping state (Figure 6).

With the increment of annealing temperature to 393 K, MP (E_f_) of G and CN-PPV–G prepared from 0.01 mg mL^−1^ solution was displaced to a much left (up) position, but their left (up) movement during higher temperature treatment (423 K) is very small (Figure 5c,d and Figure 6). Whereas for the CN-PPV–G fabricated from denser solutions (5 mg mL^−1^ and 20 mg mL^−1^), MP (E_f_) shifting tendency is reversed, a small step from 373 K to 393 K but a large one from 393 K to 423 K. For the samples from 1 mg mL^−1^ solution, MP (E_f_) changing is roughly equivalent in these two annealing processes. Within expectation, the I_ds_ order in the output curves of Figures S6c and S6d is the same to that of the transfer curves at V_g_ = 0 V in Figure 5c and Figure 5d, respectively.

Overall, it can be seen that all of CN-PPV–G are in a *n*-doping state after 393 K annealing, and the doping effect was enhanced by the annealing at higher temperature (423 K) (Figure 6). Furthermore, the *n*-doping effect demonstrates a clear correlation with the concentration of CN-PPV solution. In detail, the *n*-doping effect in the CN-PPV–G samples from dilute solutions (0.01 mg mL^−1^ and 1 mg mL^−1^) is insufficient in comparison with the samples from denser solutions (5 mg mL^−1^ and 20 mg mL^−1^), which is consistent with the results revealed by AFM and SEM, the extreme thin, even noncontinuous film in the former two samples *vs* dense and continuous one in the latter two samples. In all the conditions (before and after annealing at different temperatures), the sequence of I_ds_ values determined from the transfer curves at V_g_ = 0 V matches well with the one from output curves, indicating the high quality and reliability of our devices.

From the above analysis on the topography and electronic properties of hybrid films, it can be found that, in our tested range, the *n*-doping effect of CN-PPV to G layers can be enhanced via increment of thickness of CN-PPV part and temperature of post annealing.

### 2.3. Quick and Sensitive Detection of NO_2_ Based on CN-PPV–G Films

NO_2_ has a strong *p*-doing effect to G materials. The above tests indicate G layers can be doped to a very high *n*-doping state by CN-PPV film. Thus, CN-PPV–G hybrid films could be a very promising candidate for realizing effective detections to toxic NO_2_. The summarized MP and E_f_ positions with solution concentrations and annealing temperatures in Figure 6 imply the solution suitable for fabricating NO_2_ sensing devices should not be lower than 5 mg mL^−1^ and the annealing temperature is 423 K. To realize active sensing, NO_2_ needs diffuse into CN-PPV film rapidly. If the film is too thick, it may slow down this diffusion process, thus causing the deterioration of sensing performances. Therefore, CN-PPV–G FET for detecting NO_2_ was fabricated with a 5 mg mL^−1^ solution and annealed at 423 K.

**Figure 7:**
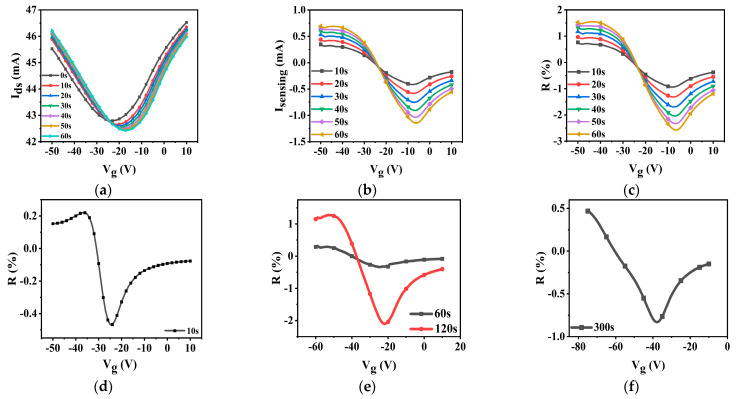
I_ds_ (**a**, V_ds_ = 1 V), I_sensing_ (**b**) and (**c**) R-V_g_ curves of CN-PPV–G transistors to 50 ppm NO_2_ in N_2_ environment; R-V_g_ curves of CN-PPV–G transistors to 25 ppm (**d**), 1 ppm (**e**) and 100 ppb (**f**) NO_2_ in N_2_ environment. 0 s means before NO_2_ exposure.

The transfer curves of CN-PPV–G transistors demonstrate immediate response to 50 ppm NO_2_ within 10 s (Figure 7a), which is an extremely high reacting speed (Table 1). NO_2_ shifts the MP of transfer curves to a right position because the *n*-doping effect is neutralized by the *p*-doping properties of NO_2_. The right displacement of MP leads to the hole (electron)-dominated conductivity apparently being increased (depressed). As a result, I_sensing_, defined as the alteration of I_ds_ with/without exposure to NO_2_ gas, is a lying-down *S* shape (Figure 7b). The data of response (R) calculated by normalizing I_sensing_ with I_ds_ of no exposure to NO_2_ follow the changing tendency of I_sensing_ (Figure 7c). Longer exposure time induces larger changing of transfer curves and higher values of I_sensing_ and R (Figure 7b,c), verifying that the alterations of the transfer curves come from the *p*-doping effect of NO_2_ indeed. Although the linear output curves of I_ds_ and I_sensing_ manifest obvious variations in NO_2_ environment as well (Figure S7), their detecting ability is not as good as that of the transfer curves due to lack of V_g_ tuning effect. As a consequence, we focused on the changing of transfer curves in the following experiments.

The concentration of NO_2_ was decreased gradually from 50 ppm to 100 ppb (Figure S8a,b and Figure 7d, 25 ppm; Figure S8c,d and Figure 7e, 1 ppm; Figure S8e,f and Figure 7f, 100 ppb). At 25 ppm of NO_2_ (Figure S8a,b and Figure 7d), our devices can still demonstrate a quick response within 10 s. The V_g_-tuned changing tendency of I_ds_, I_sensing_ and R curves is similar to the corresponding one in 50 ppm NO_2_. Decreasing the concentration of NO_2_ farther to 1 ppm (Figure S8c,d and Figure 7e), the reliable sensing signal needs to be generated in a longer time although the curve shapes remain unchanged. Longer responding time in low concentration of NO_2_ environment is reasonable because the recognizable changing of electronic properties requires accumulating enough target molecules in CN-PPV–G film. However, in comparison with the data in a previous report (Table 1), 60 s–120 s reacting time to 1 ppm NO_2_ is still a very high speed.

Successful detection of 100 ppb NO_2_ within a reasonable responding time (300 s) was realized on our CN-PPV–G FET sensors (Figure S8e,f and Figure 7f). Under this concentration, the positive peaks of I_sensing_ and R in the hole-conducting range cannot be recognized any more, but the prominent negative one originated from the depression of electron-dominated conductivity is conserved. The required detecting time for different concentration of NO_2_ was listed in Table 1, revealing the ultrahigh responding speed of our sensors in comparison with the reported devices. The reliable I_sensing_ and R curves are well above background noise although they are not designed to achieve maximum as a tradeoff of fast responding.

For NO_2_ sensors, quick responding capability is extremely important for alarming the exposure to this dangerous substance as early as possible to avoid serious damages to our health and environment in a short and long term. The reacting time of our sensors is 10 s at 25 ppm, which almost meets the demands of immediate alarming to 20 ppm NO_2_ suggested by National Institute for Occupational Safety and Health (USA) [34]. Besides the very high reacting speed, LOD of CN-PPV–G NO_2_ sensors is as low as 100 ppb (Figure S8e,f and Figure 7f), lower than 200 ppb -the highest exposure concentration of NO_2_ advised by American Conference of Governmental Industrial Hygienists [34]. These prominent sensing features indicate CN-PPV–G hybrid film is a promising candidate for developing commercial NO_2_ sensors.

**Figure 8:**
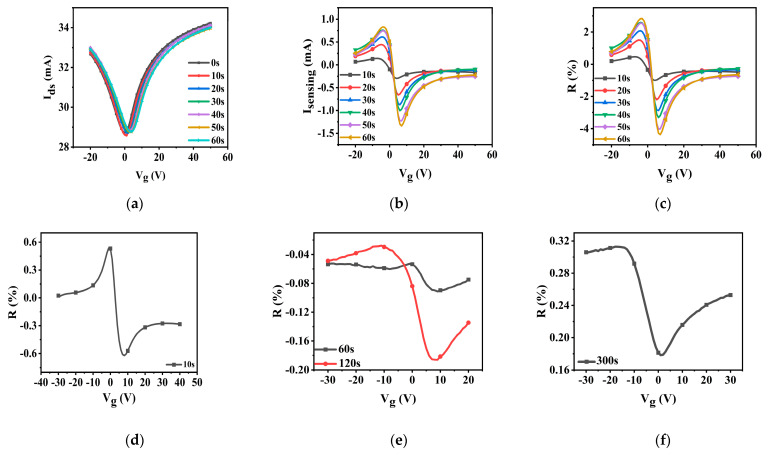
I_ds_ (**a**, V_ds_ = 1 V), I_sensing_ (**b**) and (**c**) R-V_g_ curves of CN-PPV–G transistors to 50 ppm NO_2_ in dry air; R-V_g_ curves of CN-PPV–G transistors to 25 ppm (**d**), 1 ppm (**e**) and 100 ppb (**f**) NO_2_ in dry air. 0 s means before NO_2_ exposure. NO_2_ was not introduced into the testing chamber until the transfer curves of hybrid films show negligible alterations in dry air.

Furthermore, our CN-PPV–G NO_2_ sensors exhibit excellent performances in dry air (21%O_2_/N_2_79%) environment as well. The transfer curves of I_ds_, I_sensing_ and R from 50 ppm to 100 ppb were provided in Figure 8 and Figure S9. It can be found that the main features of I_ds_, I_sensing_ and R-V_g_ curves under each concentration of NO_2_ are the same as the corresponding one in N_2_ atmosphere except the initial MP is displaced to a much right position. The *p*-doping effect of O_2_ results in the shift of the initial MP locations. NO_2_ was not introduced into the testing chamber until the transfer curves of hybrid films show negligible alterations in dry air. The remarkable operating speed and LOD of CN-PPV–G hybrids to NO_2_ are not affected by the presence of O_2_ (Table 1), unveiling the favorable characteristics of our CN-PPV–G NO_2_ sensors further.

**Figure 9:**
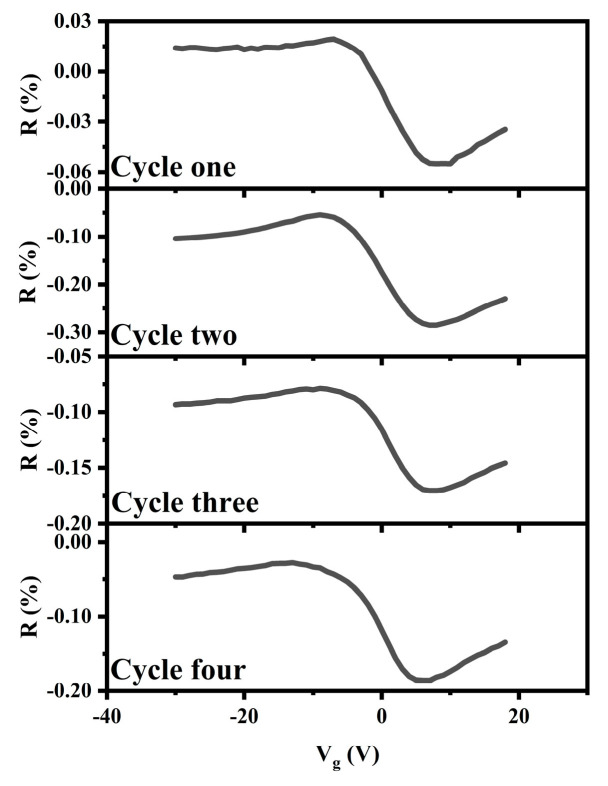
R-V_g_ (V_ds_ = 1 V) curves of CN-PPV–G transistors in the several sensing-refreshing cycles. Testing conditions, 120 s data of 1 ppm NO_2_ in dry air.

Our CN-PPV–G NO_2_ sensors can be used repeatedly (Figure 9). Adsorbed NO_2_ can be released via thermal annealing, thus, the hybrid films are refreshed. The CN-PPV–G hybrids demonstrate good stability during the sensing-refreshing cycles.

## 3. Experiments and Methods

G on Cu foil (25 μm, Alfa Aesar, Ward Hill, MA, USA) was prepared by chemical vapor deposition method using H_2_ and CH_4_. Transferring G was performed under the assistance of PMMA (poly(methyl methacrylate), 950 PMMA A4, MicroChem, Westborough, MA, USA) followed by repeatable rinsing by hot acetone and deionized water. Interdigitated Au electrodes with channel gap/width 200 μm were deposited through thermal evaporation with a steel mask after G was transferred onto SiO_2_ (300 nm)/Si chip. CN-PPV (HongKongJiSiEnBei International Trade Co., Limited, Hong Kong, China) in tetrahydrofuran (Sigma-Aldrich, Saint Louis, MO, USA, both used as received) was spin-coated on G after electrode preparation. The heating treatments of CN-PPV–G films and cooling down to room temperature were carried out in N_2_ or Ar environment.

The hybrid films were characterized with atomic force microscopy (AFM, NTEGRA Prima), scanning electron microscopy (SEM, Hitachi, Regulus 8220, Ibaraki, Japan), Raman spectroscopy (Renishaw inVia) and grazing-incident wide angle X-ray scattering (GIWAXS) experiments (1W1A Diffuse-X-ray Scattering Station, Beijing Synchrotron Radiation Facility, BSRF-1W1A, Beijing, China). The devices were tested at room temperature in a glove box or small chamber designed specifically for gas sensor investigations with an outside source meter (Keithley 2612B). The flow of gases was controlled by mass flow meter.

The Fermi level (E_f_, related to MP of the transfer curves) of CN-PPV–G samples was estimated by the V_g_ (MP) on the transfer curves and the following equation suggested in the previous reports [35,36]:

ΔE_f_ = ħ × υ_F_ × ((π × ε_0_ × ε_r_/e) × (V_g_ (MP)/d))^1/2^

ħ is reduced Planck constant; υ_F_ ≈ 10^6^ m s^−1^, is Fermi velocity of electrons in G; ε_0_ and ε_r_ (≈3.9) is the vacuum permittivity and relative permittivity of SiO_2_, respectively; e is the absolute value of electronic charge; d ≈ 300 nm, is the thickness of SiO_2_ layer.

ΔE_f_ is a positive (negative) value when V_g_ (MP) is a negative (positive) one, indicating E_f_ is above (below) the MP position.

## 4. Conclusions

In this work, the strong *n*-doping effect of CN-PPV films to G sheets is unveiled. Thick film and high-temperature post annealing can enhance the doping effect. CN-PPV films are quasi amorphous and thermal annealing has no help on forming ordered arrangement. Heavily *n*-doped CN-PPV–G transistors demonstrate very quick and sensitive response to hazardous NO_2_ gas in both N_2_ and dry air atmosphere. The *n*-doping state is reduced by the adsorption of *p*-dopant NO_2_, causing the prompt alterations of transfer curves of CN-PPV–G transistors in a short time. Relying on this mechanism, CN-PPV–G transistors can react to 25 ppm NO_2_ within 10 s, and LOD is as low as 100 ppb. These results suggest CN-PPV–G hybrid is a promising candidate for in situ monitoring the exposure of NO_2_ and triggering immediate alerts when needed. A reacting time of 10 s at 25 ppm of NO_2_ almost meets the demands of immediate alarming to 20 ppm NO_2_ suggested by National Institute for Occupational Safety and Health (USA), and 100 ppb of LOD is lower than the highest exposure concentration of NO_2_ (200 ppb) advised by American Conference of Governmental Industrial Hygienists. The remarkable detecting performances of our devices are not affected in dry air and can be restored through simple annealing treatment. This work also indicates that the transfer curves of transistors may react more promptly to the exposure of target substances and can be used to develop sensing devices designed specifically to meet the demand of quick responding whereas the smaller value of response is allowed as a tradeoff.

## Figures and Tables

**Table 1:** Response time of our CN-PPV–G sensors and the one in some previous reports.

	Environment	NO_2_ Concentration			
		50 ppm	25 ppm	1 ppm	100 ppb
Response time	N_2_	10 s	10 s	60–120 s	300 s
	Dry air	10 s	10 s	60–120 s	300 s
	References	~28 s to 25 ppm, MoSe_2_/G and ~70 s to 25 ppm, MoSe_2_, operated at RT [8]			
		~90 s to 50 ppm, Co (II) phthalocyanine/G quantum dot, operated at RT [9]			
		~300 s to 2 ppm, MoS_2_/RGO, operated at RT [20]			
		25 s to 25 ppm, PPV/porous silicon, operated at RT [28]			
		~300 s to 5 ppm, patterned G, operated above at RT (patterned G channel heated by bias voltage) [29]			
		~160 s to 5 ppm, ZnO/RGO, operated at RT [30]			
		420 s to 5 ppm, RGO, operated at RT [31]			
		~180 s to 200 ppm, GO, operated at RT [32]			
		~132 s to 50 ppm, ZnO/RGO aerogel, operated at RT [33]			

## Data Availability

Not applicable.

## Supplementary Materials

The following supporting information can be downloaded at: https://www.mdpi.com/article/10.3390/molecules28135054/s1, Figure S1: AFM topography images of CN-PPV–G films prepared with the solution of 0.01 mg mL^−1^ (**a**), 1 mg mL^−1^ (**b**) and 20 mg mL^−1^ (**c**) before and after 1 h thermal annealing at 373 K, 393 K and 423 K; Figure S2: SEM images of CN-PPV–G films prepared with the solution of 0.01 mg mL^−1^ (**a**), 1 mg mL^−1^ (**b**) and 20 mg mL^−1^ (**c**) before and after 1 h thermal annealing at 373 K, 393 K and 423 K. Contrast and brightness of zoom-in images were post adjusted to clearly show the topographical target which are not easily recognized in the small-size original micrographs; Figure S3: (**a**,**b**) GIWAXS patterns collected on CN-PPV–G films prepared from 5 mg mL^−1^ before and after annealed 1 h at 373 K and 393 K, respectively. The first dot in the z direction of each image is not a signal that can be reasonably generated by our CN-PPV–G films. So we discarded the corresponding peak in the fitting profiles (Figure S4); Figure S4: (**a**–**d**) The z direction fitting profiles of CN-PPV–G films prepared from 5 mg mL^−1^ before and after annealed 1 h at 373 K, 393 K and 423 K, respectively. In each profile, the first peak produced by the first dot in GIWAXS pattern is discarded because the first dot is not a signal that can be reasonably generated by our CN-PPV–G films; Figure S5: Raman spectroscopy of CN-PPV–G casted from 0.01 mg mL^−1^ solution after annealing 1 h at 423 K; Figure S6: The output curves of pristine G and CN-PPV–G samples casted from different concentrations before (**a**) and after annealing 1 h at 373 K (**b**), 393 K (**c**) and 423 K (**d**); Figure S7: I_ds_ (**a**) and I_sensing_ (**b**) -V_ds_ curves of CN-PPV–G transistors exposure to 50 ppm NO_2_ in N_2_ environment at V_g_ = 0 V. 0 s means before NO_2_ exposure; Figure S8: I_ds_ (**a**, V_ds_ = 1 V) and I_sensing_ (**b**)-V_g_ curves of CN-PPV–G transistors to 25 ppm NO_2_ in N_2_ environment; I_ds_ (**c**, V_ds_ = 1 V) and I_sensing_ (**d**)-V_g_ curves of CN-PPV–G transistors to 1 ppm NO_2_ in N_2_ environment; I_ds_ (**e**, V_ds_ = 1 V) and I_sensing_ (**f**)-V_g_ curves of CN-PPV–G transistors to 100 ppb NO_2_ in N_2_ environment. 0 s mneans before NO_2_ exposure; Figure S9: I_ds_ (**a**, V_ds_ = 1 V) and I_sensing_ (**b**)-V_g_ curves of CN-PPV–G transistors to 25 ppm NO_2_ in dry air; I_ds_ (**c**, V_ds_ = 1 V) and I_sensing_ (**d**)-V_g_ curves of CN-PPV–G transistors to 1 ppm NO_2_ in in dry air; I_ds_ (**e**, V_ds_ = 1 V) and I_sensing_ (**f**)-V_g_ curves of CN-PPV–G transistors to 100 ppb NO_2_ in dry air. 0 s mneans before NO_2_ exposure. NO_2_ was not introduced into the testing chamber until the transfer curves of hybrid films show negligible alteractions in dry air.
