# Supplementary material for: Enabling Quick Response to Nitrogen Dioxide at Room Temperature and Limit of Detection to Ppb Level by Heavily n-Doped Graphene Hybrid Transistor
Source: Molecules. 2023 Jun 28;28(13):5054. doi: 10.3390/molecules28135054 (PMC10343478; doi:10.3390/molecules28135054)
Supplement: Supplementary file 1 [file molecules-28-05054-s001.zip › molecules-2393452-supplementary.pdf]

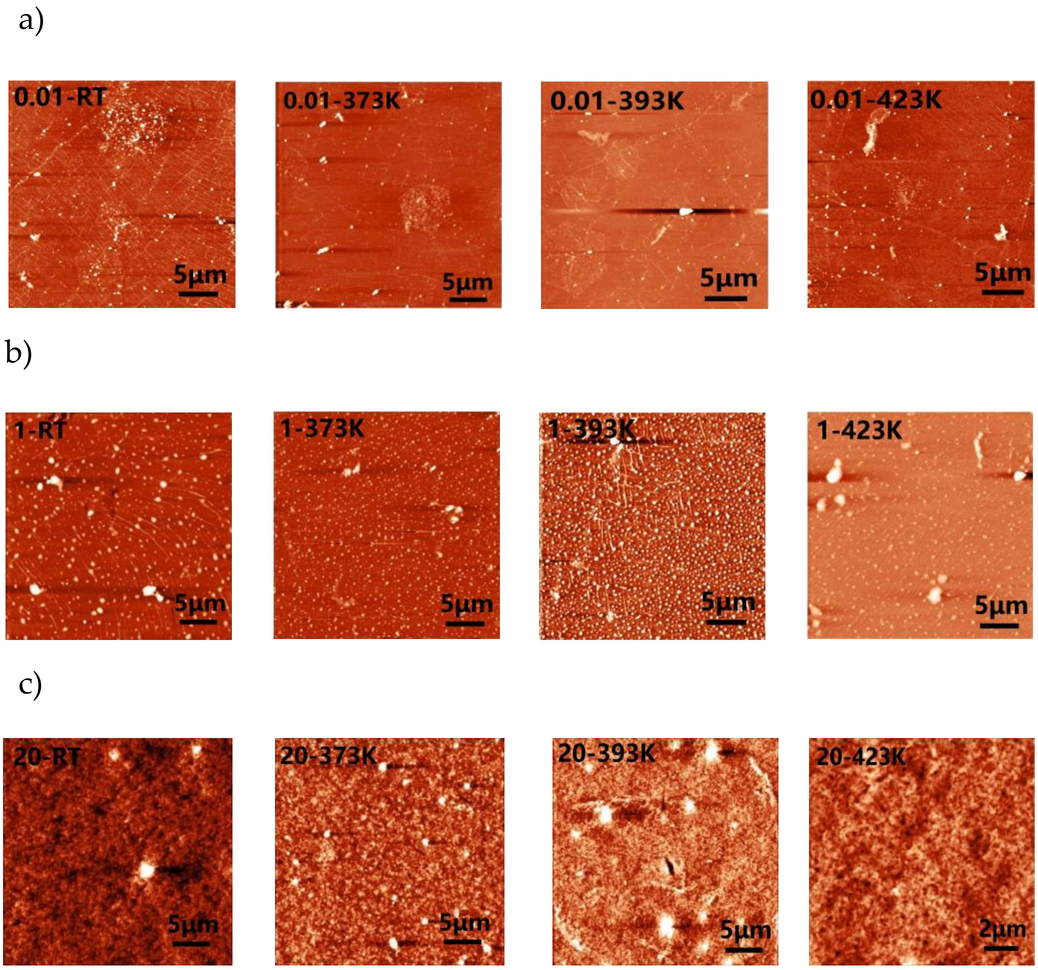

Figure S1. AFM topography images of CN-PPV–G films prepared with the solution of 0.01 mg mL^{-1} (**a**), 1 mg mL^{-1} (**b**) and 20 mg mL^{-1} (**c**) before and after 1 h thermal annealing at 373 K, 393 K and 423 K.

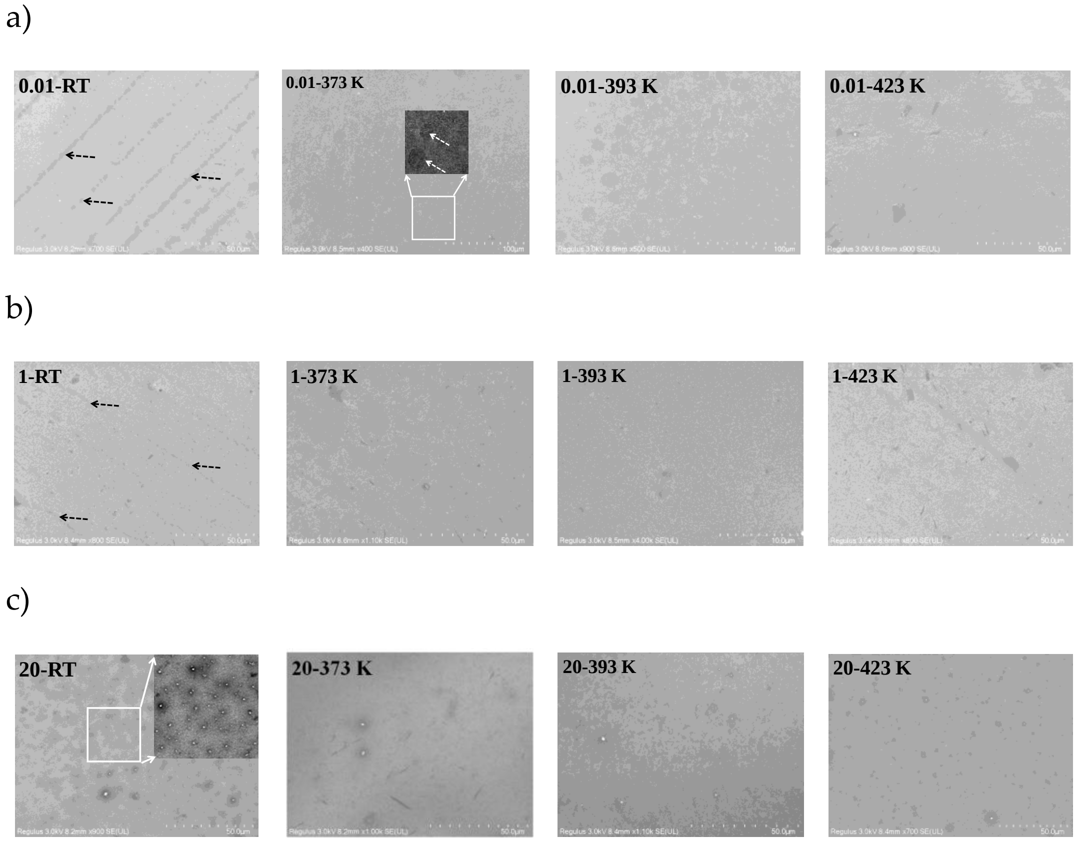

Figure S2. SEM images of CN-PPV–G films prepared with the solution of 0.01 mg mL^{-1} (**a**), 1 mg mL^{-1} (**b**) and 20 mg mL^{-1} (**c**) before and after 1 h thermal annealing at 373 K, 393 K and 423 K. Contrast and brightness of zoom-in images were post adjusted to clearly show the topographical target which are not easily recognized in the small-size original micrographs.

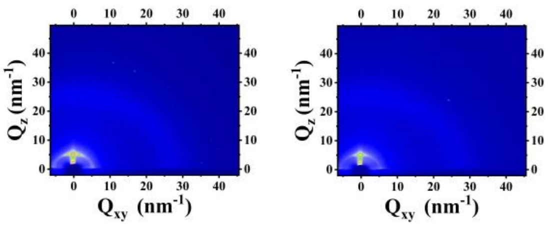

a) b)

Figure S3. (**a**) and (**b**) GIWAXS patterns collected on CN-PPV–G films prepared from 5 mg mL^{-1} before and after annealed 1 h at 373 K and 393 K, respectively. The first dot in the *z* direction of each image is not a signal that can be reasonably generated by our CN-PPV–G films. So we discarded the corresponding peak in the fitting profiles (Figure S4).

a) b) c) d)

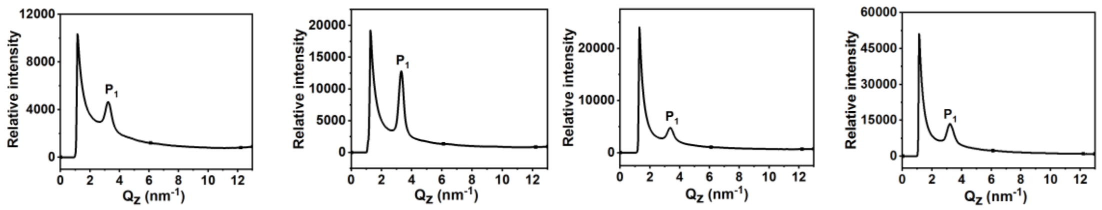

Figure S4. (**a–d**) The *z* direction fitting profiles of CN-PPV–G films prepared from 5 mg mL^{-1} before and after annealed 1 h at 373 K, 393 K and 423 K, respectively. In each profile, the first peak produced by the first dot in GIWAXS pattern is discarded because the first dot is not a signal that can be reasonably generated by our CN-PPV–G films.

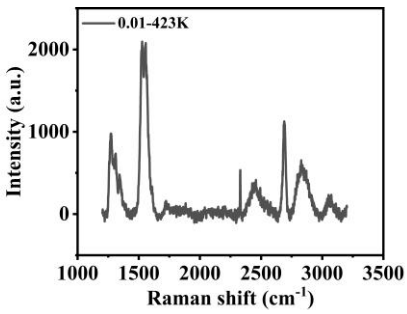

Figure S5. Raman spectroscopy of CN-PPV–G casted from 0.01 mg mL^{-1} solution after annealing 1 h at 423 K.

a) b) c) d)

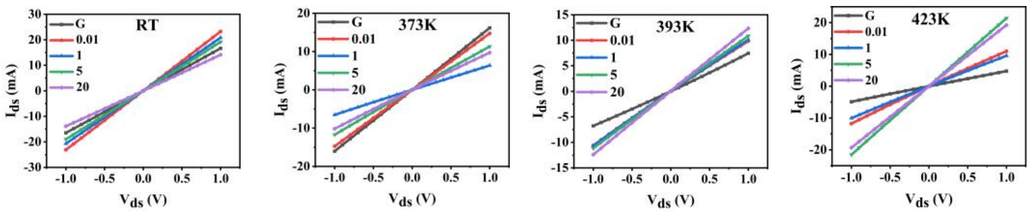

Figure S6. The output curves of pristine G and CN-PPV–G samples casted from different concentrations before (**a**) and after annealing 1 h at 373 K (**b**), 393 K (**c**) and 423 K (**d**).

a) b)

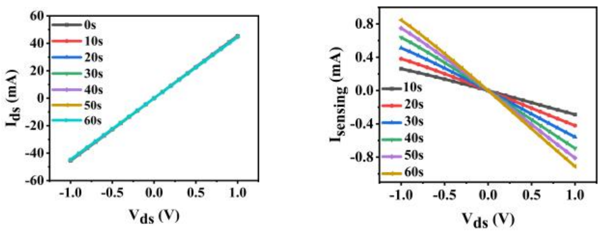

Figure S7. I_{ds} (**a**) and $I_{sensing}$ (**b**) -V_{ds} curves of CN-PPV–G transistors exposure to 50 ppm NO_2 in N_2 environment at V_g = 0 V. 0 s means before NO_2 exposure.

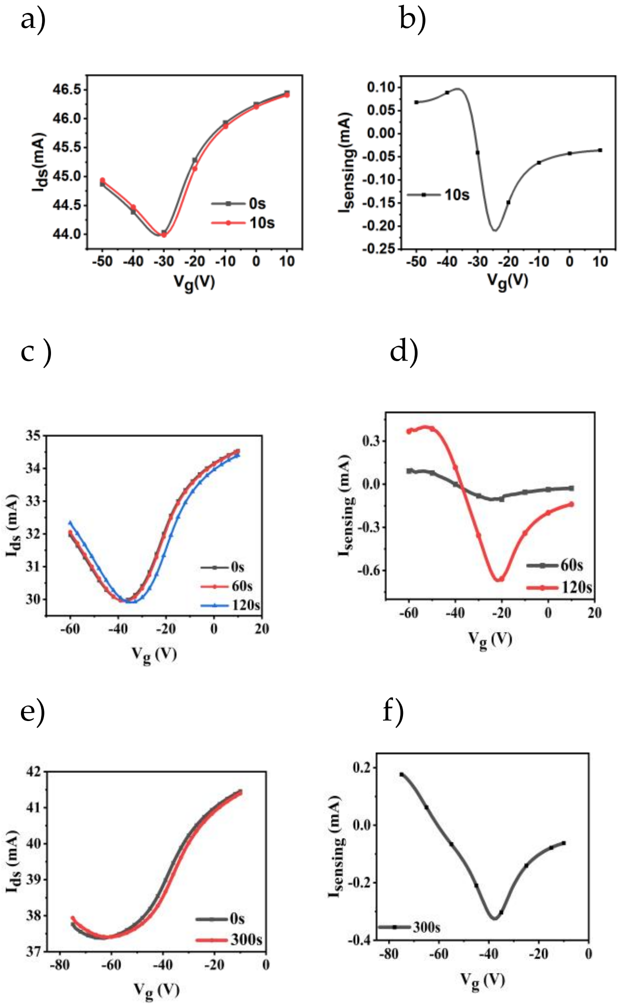

Figure S8. I_{ds} (**a**, V_{ds}=1 V) and $I_{sensing}$ (**b**)-V_g curves of CN-PPV–G transistors to 25 ppm NO_2 in N_2 environment; I_{ds} (**c**, V_{ds}=1 V) and $I_{sensing}$ (**d**)-V_g curves of CN-PPV–G transistors to 1 ppm NO_2 in N_2 environment; I_{ds} (**e**, V_{ds}=1 V) and $I_{sensing}$ (**f**)-V_g curves of CN-PPV–G transistors to 100 ppb NO_2 in N_2 environment. 0 s mneans before NO_2 exposure.

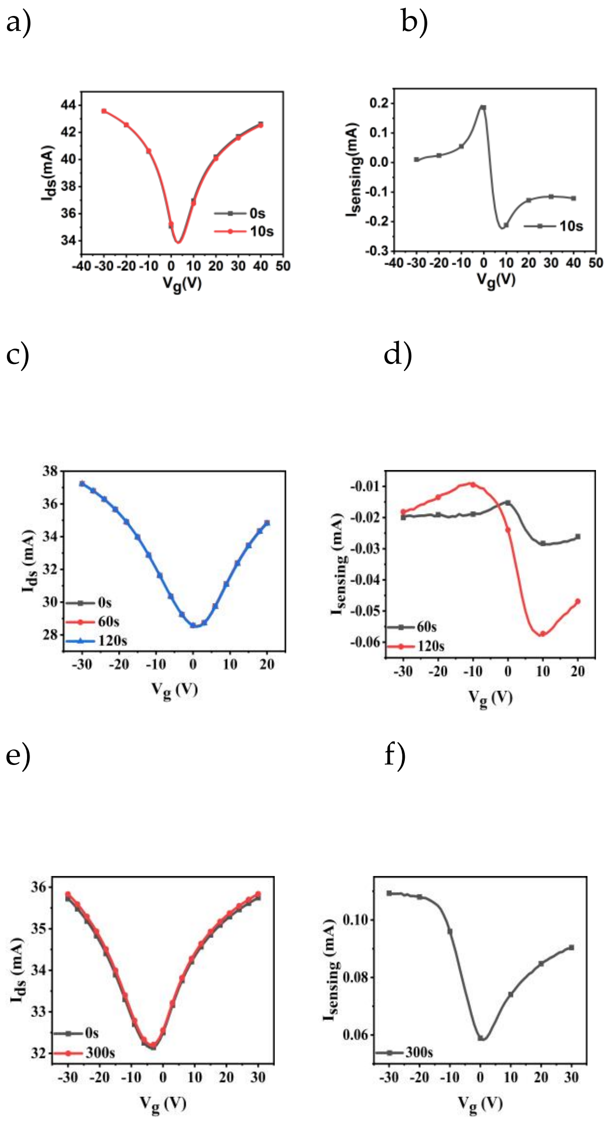

Figure S9. I_{ds} (**a**, V_{ds}=1 V) and $I_{sensing}$ (**b**)-V_g curves of CN-PPV–G transistors to 25 ppm NO_2 in dry air; I_{ds} (**c**, V_{ds}=1 V) and $I_{sensing}$ (**d**)-V_g curves of CN-PPV–G transistors to 1 ppm NO_2 in in dry air; I_{ds} (**e**, V_{ds}=1 V) and $I_{sensing}$ (**f**)-V_g curves of CN-PPV–G transistors to 100 ppb NO_2 in dry air. 0 s mneans before NO_2 exposure. NO_2 was not introduced into the testing chamber until the transfer curves of hybrid films show negligible alteractions in dry air.
